# Severe Pressure Ulcers in Two Patients With Adrenoleukodystrophy

**DOI:** 10.7759/cureus.37669

**Published:** 2023-04-17

**Authors:** Koji Obara

**Affiliations:** 1 Neurology, National Hospital Organization Akita National Hospital, Yurihonjo, JPN

**Keywords:** pressure ulcer, incontinence, immobility, adrenoleukodystrophy, abcd1

## Abstract

Adrenoleukodystrophy (ALD) is a rare X-linked disease that affects the metabolism of very long-chain fatty acids (VLCFAs), leading to cognitive deterioration, progressive spastic paraplegia, sensory disturbance, adrenocortical insufficiency, and bladder and bowel abnormalities. Although the symptoms of ALD correspond to the risk of developing pressure ulcers, a pressure ulcer has never been listed as a complication of ALD. We present two cases of ALD with severe pressure ulcers in the pelvic region and feet. The first case was a 27-year-old male patient with adolescent cerebral-type ALD who had pressure ulcers with bone exposure on the sacral and bilateral greater trochanter region. The second case was a 64-year-old male patient with adrenomyeloneuropathy (AMN) phenotype who had pressure ulcers on the sacral region and both feet. Both patients had VLCFA accumulation and a likely pathogenic variant in the *ABCD1* gene, the causative gene of ALD. These cases indicate that ALD patients with immobility and incontinence have a higher risk of developing severe pressure ulcers, which requires the proactive identification of ALD patients and early multidisciplinary intervention for patients and their families to prevent the development of pressure ulcers.

## Introduction

Adrenoleukodystrophy (ALD) is a rare X-linked disease that affects the metabolism of very long-chain fatty acids (VLCFAs) [1,2]. This disease results from a pathogenic variant in the *ABCD1* gene that encodes a peroxisomal transporter protein and affects the nervous system and the adrenal gland [1,2]. ALD has four primary phenotypes: cerebral ALD, adrenomyeloneuropathy (AMN), Addison's disease, and female ALD. Patients with ALD show cognitive deterioration, progressive spastic paraplegia, sensory disturbance, adrenocortical insufficiency, and bladder and bowel abnormalities, leading to immobility with incontinence [1,2]. These symptoms of ALD correspond to the risk of developing pressure ulcers, but a pressure ulcer has never been listed as a complication of ALD. Herein, we report two patients with ALD who showed immobility and incontinence and developed severe pressure ulcers in the pelvic region and feet.

## Case presentation

**Figure 1:**
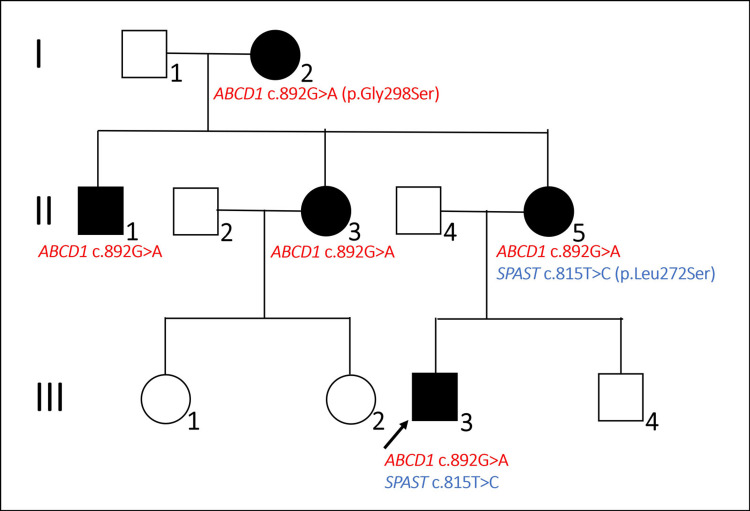
Pedigree of the patient's family A black symbol indicates patients with the following ALD phenotypes: cerebral ALD, AMN, and female ALD, all of which carry the p.Gly298Ser variant in the *ABCD1 *gene (red letters). Patient 1 is III-1 and proband (black arrow). Patient 2 is II-1. Patient 1 and his mother (II-5) also have the p.Leu272Ser variant in the *SPAST* gene (blue letters) ALD, Adrenoleukodystrophy; AMN, adrenomyeloneuropathy

Figure 1 shows the family pedigree of the patients.

Patient 1

**Figure 2:**
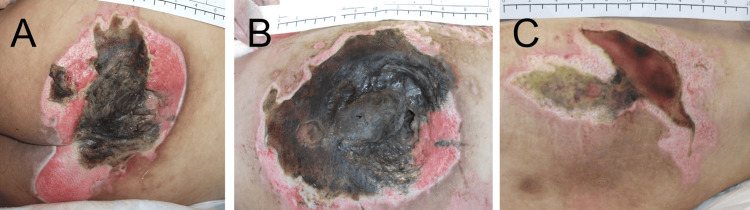
Pressure ulcers in patient 1 Pressure ulcers with necrotic tissue on (A) the sacral, (B) the left greater trochanter, and (C) the right greater trochanter regions

A 27-year-old male patient (III-3) urgently visited our institute with lightheadedness and a high fever. His grandmother, aunt, uncle, and mother had gait problems starting in their 40s. Three years before this visit of patient 1, his mother's (II-5) p.Leu272Ser variant in the *SPAST* gene, a causative gene of spastic paraplegia type 4 (SPG4), was identified. At 16, he noted an onset of spasticity in the legs. At 18, he became unable to run and noticed urinary incontinence. By the age of 20, he had difficulty walking without assistance. Synchronously with these symptoms, he gradually became apathetic and mute. As a result, he dropped out of college. Later, he isolated himself in his room all day and night. A physical examination revealed a temperature of 39.4°C, a pulse of 129 beats/minute, a breath rate of 26 times/minute, a blood pressure of 73/51 mmHg, and deep pressure ulcers with black necrotic tissue with associated abscess formation on his sacral and bilateral greater trochanter region (Figure 2).

On neurological examination, he was alert but apathetic. He had dysarthria and hardly talked. Cranial nerves appeared intact except for dysarthria. He could not hold a sitting position without assistance. He was hypertonic in all four extremities and has severely limited extension of the hip, knee, and ankle joints by contractures. The Medical Research Council muscle grading scale was 3 or 4/5 for the upper extremities and 1/5 for the lower extremities. The finger-nose-finger test revealed mild oscillation and dysmetria without laterality. All modalities of sensation were severely decreased in bilateral lower extremities. His muscle stretch reflexes were hyperactive in the extremities with knee and ankle clonus, and the plantar responses were extensor. He displayed urinary and fecal incontinence. Laboratory testing revealed a severe inflammatory response with elevated procalcitonin and hypoalbuminemia (Table 1).

**Table 1:** Laboratory testing of patient 1 ACTH: adrenocorticotropic hormone

Test	Results	Reference range
On admission		
White cell count (per μL)	19,370	3,300-8,600
Neutrophil relative (%)	91.8	40.5-72.1
Red cell count (per μL)	322 × 10^4^	435-555 × 10^4^
Hemoglobin (g/dL)	9.0	13.7-16.8
Platelets count (per μL)	46.0 × 10^4^	15.8-34.8 × 10^4^
Fibrinogen (mg/dL)	650	170-410
Aspartate aminotransferase (U/L)	49	13-30
Alanine aminotransferase (U/L)	28	10-42
Alkaline phosphatase (U/L)	337	106-322
Urea nitrogen (mg/dL)	16.9	8.0-20.0
Creatinine (mg/dL)	0.71	0.46-0.79
Creatine phosphokinase (U/L)	262	41-153
Total protein (g/dL)	4.6	6.6-8.1
Albumin (g/dL)	1.5	4.1-5.1
Sodium (mmol/L)	129	138-145
Potassium (mmol/L)	3.3	3.6-4.8
Chloride (mmol/L)	96	101-108
Calcium (mg/dL)	6.9	8.8-10.1
C-reactive protein (mg/dL)	22.7	0-0.14
Vitamin B12 (pg/mL)	202	233-914
Folate (ng/mL)	4.3	3.6-12.9
Procalcitonin (ng/mL)	8.27	≤0.05
During the course		
ACTH (pg/mL)	135.2	7.2-63.3
Cortisol (μg/dL)	2.0	4.5-21.1
C26/C22 ratio of very long-chain fatty acid	0.072	0.007-0.017

**Figure 3:**
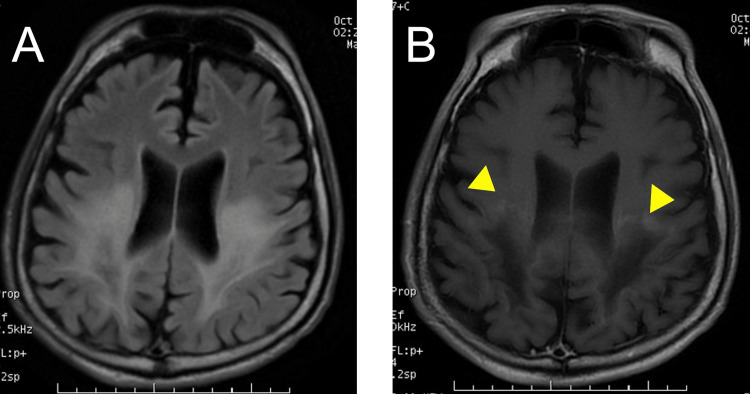
Brain MRI findings in patient 1 (A) Fluid-attenuated inversion recovery (FLAIR) imaging shows a diffuse high-intensity lesion in deep white matter in the parieto-occipital lobes. (B) Contrast-enhanced T1-weighted imaging shows border enhancement in the anterior periphery of the lesion demonstrated by the FLAIR image (yellow arrowheads)

Both blood and skin and soft tissue cultures grew *Proteus mirabilis*, which was sensitive to beta-lactams. After admission, we treated him with infusion, norepinephrine, and meropenem, and he underwent surgical debridement of extensive necrotic tissue from the ulcers. Nine months after the admission, the patient developed septic arthritis of the left hip joint and underwent two arthrotomies with drainage and curettage. Over the next 13 months, ulcers on the sacral and bilateral greater trochanter region became scar tissues, and the left hip joint showed bony ankylosis. Thereafter, small ulcers often occurred in the same areas and were intractable. During that time, his cognitive function declined further, and he became unresponsive to three of eight external stimuli. At the age of 27, a brain MRI showed symmetric lesions with a high signal in the posterior deep white matter on T2-weighted imaging (T2WI) and fluid-attenuated inversion recovery (FLAIR) imaging. A repeat brain MRI showed a posterior cerebral white matter lesion enlargement and slight enhancement with a gadolinium contrast agent at the margins of the white matter lesion (Figure 3).

Therefore, we considered the possibility of ALD based on contrast-enhanced cerebral white matter lesions. Additional laboratory tests showed elevated adrenocorticotropic hormone (ACTH), low cortisol, and elevated C26/C22 ratio of VLCFA (Table 1).

After obtaining written consent from the patient's family, we performed genetic testing of the *ABCD1 *gene using next-gene sequencing (NGS) (Illumina MiSeq-based method and VariantStudio analysis) and genomic DNA extracted from his white blood cells. Next, we confirmed the proposed variant by NGS via the Sanger sequencing of the DNA collected from the patient and his grandmother, aunt, uncle (patient 2), and mother. Moreover, we performed the Sanger sequencing of exon 5 of the *SPAST* gene in the patient and his grandmother, aunt, uncle (patient 2), and mother. As a result, we identified a heterozygous or hemizygous variant in exon 1 of the *ABCD1* gene (NM_000033.4:c.892G>A {p.Gly298Ser}) in the patient and his grandmother, aunt, uncle (patient 2), and mother and a heterozygous variant in exon 5 of the *SPAST* gene (NM_014946.4:c.815T>C {p.Leu272Ser}) in the patient and his mother (Figure 1). His grandmother, uncle, and aunt did not have this variant in exon 5 of the *SPAST* gene. The p.Gly298Ser variant in the *ABCD* gene was previously registered in ClinVar as likely pathogenic (accession: SCV002045762.1 and SCV000964982.2). On the other hand, we did not find the p.Leu272Ser variant in the *SPAST* gene in the following genetic public database: Human Gene Mutation Database (www.hgmd.cf.ac.uk/ac/index.php), Leiden Open Variation Database (LOVD), National Center for Biotechnology Information (NCBI) ClinVar, Single Nucleotide Polymorphism Database (dbSNP), and Genome Aggregation Database (gnomAD). Polymorphism phenotyping 2 (PolyPhen-2) and sorting intolerant from tolerant (SIFT) predicted that this variant in the *SPAST* gene was benign and tolerated, respectively. Finally, we attributed his symptoms to ALD and diagnosed him with adolescent cerebral-type ALD. At the age of 31 years, he passed away due to respiratory failure caused by bronchopneumonia.

Patient 2

**Figure 4:**
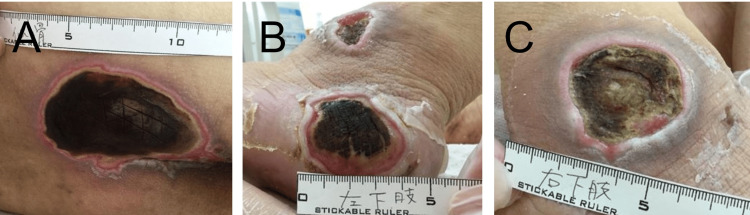
Pressure ulcers in patient 2 Pressure ulcers with necrotic tissue on (A) the sacral, (B) the left lateral malleus and lateral heel, and (C) the right lateral malleus regions

A 64-year-old male patient (II-1) visited our institute with a high fever and symptoms of dehydration for three days. He noted an onset of spasticity in the legs in his teens. Since his mid-30s, he could not run and noticed pollakiuria and urinary incontinence. He worked as a healthcare worker until he was 55. At 58, he began to require a wheelchair, and red macules repeatedly appeared on the bony prominences of his sacral region. At 63, because his father, his primary caretaker, had died, he tended to be bedridden except for weekly home care services and had poor nutrition and hygiene. As a result, his legs and feet became severely edematous. Two months before admission, bullae of 5 cm in diameter developed on both heels. Physical examination revealed a temperature of 39.2°C, a pulse of 120 beats/minute, a breath rate of 20 times/minute, a blood pressure of 120/90 mmHg, and deep pressure ulcers with a black necrotic tissue on his sacral, bilateral lateral malleus, and left lateral heel region (Figure 4).

The bullae on his bilateral heels ruptured, and the bottoms showed dark red erosions. In addition, small dark pigmentations dotted the lips on his face. On neurological examination, cognition and speech were normal. Cranial nerves were intact. He could not stand, walk, or sit without assistance. He had hypertonus in the lower extremities and slightly limited extension of the hip, knee, and ankle joints by contractures. The Medical Research Council muscle grading scale was 5/5 for the upper extremities and 1/5 for the lower extremities. The finger-nose-finger test did not reveal ataxia. All modalities of sensation were severely decreased in bilateral lower extremities. His muscle stretch reflexes were hyperactive at both knees and absent at the ankles, and the plantar responses were extensor. Additionally, he suffered from urinary and fecal incontinence. Laboratory testing showed an inflammatory response and hypoalbuminemia (Table 2). ACTH and cortisol were within normal limits, while the C26/C22 ratio of VLCFA was elevated (Table 2).

**Table 2:** Laboratory testing of patient 2 ACTH: adrenocorticotropic hormone

Test	Results	Reference range
On admission		
White cell count (per μL)	12,400	3,300-8,600
Neutrophil relative (%)	85.0	40.5-72.1
Red cell count (per μL)	410 × 10^4^	435-555 × 10^4^
Hemoglobin (g/dL)	11.9	13.7-16.8
Platelets count (per μL)	42.4 × 10^4^	15.8-34.8 × 10^4^
Aspartate aminotransferase (U/L)	45	13-30
Alanine aminotransferase (U/L)	31	10-42
Alkaline phosphatase (U/L)	346	106-322
Urea nitrogen (mg/dL)	19.6	8.0-20.0
Creatinine (mg/dL)	0.77	0.46-0.79
Creatine phosphokinase (U/L)	221	41-153
Total protein (g/dL)	6.2	6.6-8.1
Albumin (g/dL)	2.1	4.1-5.1
Sodium (mmol/L)	136	138-145
Potassium (mmol/L)	4.1	3.6-4.8
Chloride (mmol/L)	100	101-108
Calcium (mg/dL)	8.2	8.8-10.1
C-reactive protein (mg/dL)	13.2	0-0.14
ACTH (pg/mL)	56.6	7.2-63.3
Cortisol (μg/dL)	8.8	4.5-21.1
C26/C22 ratio of very long-chain fatty acid	0.057	0.007-0.017

**Figure 5:**
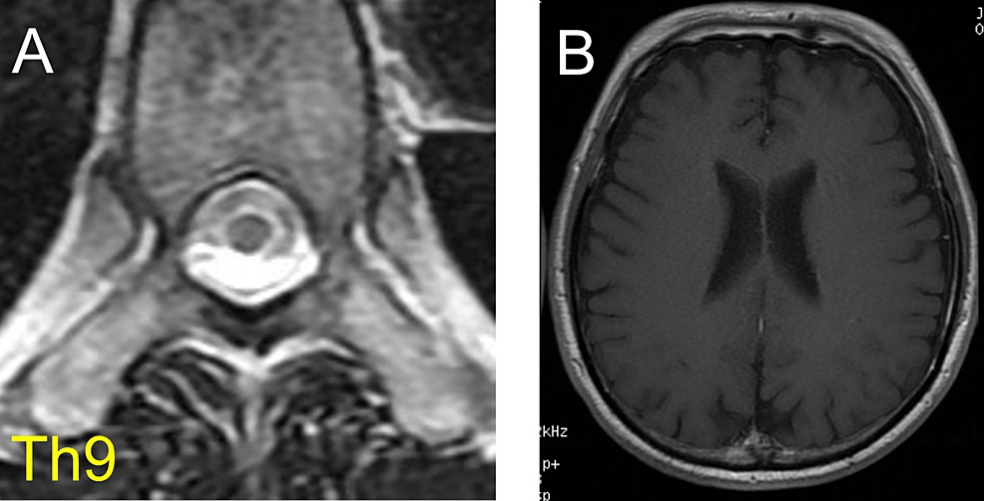
Spinal and brain MRI findings in patient 2 (A) A spinal MRI shows the atrophy of the thoracic cord on the ninth thoracic vertebra level. (B) On brain MRI, contrast-enhanced T1-weighted imaging does not show enhanced lesions and atrophy

A spinal MRI showed the atrophy of the thoracic and lumber spinal cord (Figure 5A). A brain MRI showed normal findings with no contrast-enhancing lesions (Figure 5B).

After admission, we treated him with meropenem, and he underwent surgical debridement of extensive necrotic tissue from the ulcers. After about three months, the ulcers on both feet were epithelialized. On the other hand, the ulcer in the sacral region did not heal sufficiently, so we performed negative pressure wound therapy (NPWT) via the RENASYS TOUCH™ vacuum pump (Smith & Nephew, Hull, United Kingdom), and the ulcer was finally epithelialized eight months after admission. A nerve conduction study was performed following the healing of his pressure ulcers and revealed an absence of the compound motor action potential and sensory nerve action potential of the lower extremities. As noted above, he had the p.Gly298Ser variant in the *ABCD1* gene but did not have the p.Leu272Ser variant in exon 5 of the *SPAST* gene. Finally, we diagnosed him with AMN.

## Discussion

We found two crucial clinical points. First, severe pressure ulcers developed in two patients with ALD. A pressure ulcer (decubitus and bedsore) is one of the most devastating complications in patients with various neurological diseases [3,4]. It is a nonspecific disease, although it has been reported more frequently in spinal cord injury patients [5]. In our search of English articles in PubMed, we found no article describing pressure ulcers as a complication of ALD. However, ALD causing the involvement of the central and peripheral nervous system with bladder and bowel abnormalities may carry a high risk of pressure ulcer development. We hypothesized that the following factors contributed to the development of pressure ulcers in the two patients: immobility due to progressive ALD, poor nutrition, urinary and fecal incontinence, and a lack of pain perception and expression due to cerebral and spinal involvement. In particular, withdrawal due to cognitive deterioration and psychiatric symptoms in patient 1 and inadequate caregiving capacity in patient 2 were thought to exacerbate their immobility. In addition, patient 2 also had electrophysiologic confirmed peripheral sensory neuropathy in the lower extremities, which may have contributed to the development of pressure ulcers. However, we could not verify this for patient 1. As in the patients we presented, a severe pressure ulcer can be associated with bacteremia, cellulitis, osteomyelitis, and septic arthritis, among others, and be life-threatening [6]. Moreover, even if it heals, orthopedic sequelae may remain, leading to further immobility and quality of life deterioration.

The second crucial clinical point is that patient 1 and his mother had a *SPAST* and *ABCD* genes variant. Although the significance of the p.Leu272Ser variant in the *SPAST* gene was unknown, we initially diagnosed them with SPG4 due to the *SPAST* variant, which delayed the diagnosis of ALD and corticosteroid replacement therapy. Later, in silico analyses predicted that the p.Leu272Ser variant in the *SPAST* gene is benign and tolerated. SPG4 is the highest frequency and accounts for 15%-40% of all autosomal dominant hereditary spastic paraplegias (ADHSPs) [7]. In middle age, patients with SPG4 show slowly progressive gait disturbance and usually cannot walk without assistance [7]. Although SPG4 usually shows a pure hereditary spastic paraplegia (HSP) form, it also can cause urinary incontinence and sensory disorder [7]. Therefore, it may be difficult to differentiate male AMN and female ALD from pure HSP, including SPG4, by clinical symptoms alone. Although no male-to-male transmission, skin pigmentation, high ACTH value, and the ratio of VLCFA suggest ALD, an analysis of the *ABCD* gene may finally need to differentiate it from HSPs.

## Conclusions

Severe pressure ulcers developed in two patients with ALD; patient 1 and his mother had a variant in the *SPAST* and *ABCD* genes. Patients with ALD may be at high risk of developing pressure ulcers and their severity. Proactively identifying ALD patients and early multidisciplinary intervention for patients and their families are needed to prevent the development of pressure ulcers.
